# RNA干扰文库的研究进展及其在肿瘤研究中的应用

**DOI:** 10.3779/j.issn.1009-3419.2013.02.08

**Published:** 2013-02-20

**Affiliations:** 150001 哈尔滨，哈尔滨医科大学医学院附属第三临床学院肿瘤内科 Department of Medical Oncology, the Third Affiliated Hospital of Harbin Medical University, Harbin 150001, China

**Keywords:** RNAi, RNAi文库, 肿瘤, 应用, RNAi, RNAi library, Tumor, Application

## Abstract

RNA干扰（RNA interference, RNAi）是由双链RNA引发的同源mRNA特异性降解的现象，RNAi文库是利用RNAi技术人工构建的用来筛选表型的混合文库。由于RNAi文库在基因研究领域取得了重大突破，目前已广泛地应用于医学研究领域尤其是肿瘤研究领域中。本文综述了RNAi文库的研究进展，并对RNAi文库在肿瘤研究方面的应用进行概述。

## Abstract (en)

RNA interference is a homologous mRNA special degradation phenomenon which is caused by the double-stranded RNA. RNAi library is a pooled library that is artificially constructed using RNAi technology. As RNAi library has made a major breakthrough in the field of genetic research, it has been widely used in the field of medical research, especially in the field of cancer research. This review discussed the research progress of RNAi library and its applications in cancer research.

在研究人员发现RNA干扰（RNA interference, RNAi）技术不久后，在无脊椎动物、植物及哺乳动物中，用于全基因组范围内遗传筛选的RNAi文库迅速发展起来，历经了从早期的化学合成RNAi文库到慢病毒RNAi文库各个阶段。由于RNAi文库无法比拟的优越性，目前已广泛应用于肿瘤研究中。本文将从不同RNAi文库的优越性和局限性以及在肿瘤研究中的应用加以论述。

## 1 RNA干扰文库的概念和发展

1970年分子生物学中心法则的定义使人们发现反义碱基配对可干扰基因表达，可最终应用于疾病的机理研究和治疗^[1]^。1993年，人们发现一类非编码微小RNA（microRNA, miRNA）在真核细胞中充当主要的调控子^[2]^，加速了RNA研究的发展。1998年，第一个反义的药物——福米韦生（Fomivirsen），被食品和药物管理局（Food and Drug Administration, FDA）批准治疗巨细胞病毒视网膜炎^[3]^，与此同时，研究者在新秀丽小杆线虫中发现了RNAi机制^[4]^，并因此荣获2006年诺贝尔经济学奖。RNAi的发现改变了基因调控的面貌，它在基因功能研究和疾病治疗上也取得了新进展^[5]^。

RNAi文库是人工构建的能通过诱导RNAi抑制众多不同基因表达的混合文库，可以用于建立功能缺陷（loss of function）的生物或细胞库，进行表型的筛选。2000年RNAi文库的产生是RNAi研究的一个重要飞跃^[6, 7]^，其中在全基因组范围内进行功能缺失的筛选，优于之前采用的候选基因的方法。研究者利用RNAi文库发现了许多与生物功能有关的新基因和治疗疾病的新靶点，在基因功能研究领域迈出了重大的一步。在无脊椎动物^[4]^和植物^[8]^中，RNAi在转录后水平的基因沉默首先通过导入长的双链RNA（double-stranded RNA, dsRNA），通常为几百个核苷酸的长度，随后进行宿主基因沉默/miRNA机制加工成功能性的小干扰RNA（small interference RNA, siRNA），通常为21个-25个核苷酸的长度。哺乳动物的RNAi由siRNA触发：siRNA可以经化学合成后或由载体传递导入细胞，常用的载体有表达短发夹RNA（short hairpin RNA, shRNA）前体的病毒载体^[9]^。SiRNA进入细胞后可以结合形成RNA诱导沉默复合体（RNA-induced silencing complex, RISC），siRNA打开双链激活RISC，激活的RISC通过碱基互补配对与对应的mRNA结合并进行降解。基于其不同的加工处理机制，研究者已经创建了各种类型的包括无脊椎动物、植物和哺乳动物的RNAi文库。我们将全面探讨RNAi文库的发展：从早期的化学合成RNAi文库到最近的慢病毒RNAi文库，并对RNAi文库在肿瘤研究方面的应用进行概述。

## 2 适用于不同对象的RNAi文库

### 2.1 适用于无脊椎动物和植物的dsRNA文库

高通量RNAi筛选最初出现在新秀丽小杆线虫中^[6, 7]^，指某一基因被沉默之后表型的改变。2000年一个针对近90%位于线虫染色体Ⅰ上已知基因的由细菌表达的长dsRNA文库（2, 445个独立克隆）被首次提出^[6]^，这是一个可重复使用的文库，只需将细菌的克隆供给蠕虫，便可以实现无限制的RNAi筛选，但它仍然只是RNAi文库的雏形。在几乎同一时间，一个针对线虫第三染色体上23, 000个开放阅读框架的dsRNA库被开发出来^[7]^。文库构建的过程是首先对单个开放阅读框进行PCR扩增，产生DNA模板，然后在体外转录产生dsRNA。最后，将这些dsRNA注入蠕虫中进行功能分析就可以实现大规模筛选。不久，这些文库扩大到覆盖98%的线虫基因组的已知基因^[10]^。类似的方法也适用于构建针对91%果蝇已知基因的dsRNA文库^[11]^。但若应用于哺乳动物细胞中，长dsRNA直接转染会引起细胞毒性反应，基因沉默效果差，因此不适用于哺乳动物细胞。

### 2.2 适用于无脊椎动物、植物和哺乳动物的短发夹RNA（shRNA）文库

RNA干扰的另一个手段是载体（质粒）介导的shRNA的转基因表达。shRNA能被细胞内的siRNA或miRNA处理为功能性的siRNA。RNA聚合酶Ⅲ启动子，如H1或U6启动子能启动传统的shRNA（相当于miRNA的前体）的表达。在果蝇中，传统shRNA方法已被广泛使用，以产生转基因RNAi株系并在这个物种的所有体细胞组织中应用。第二代shRNA，也称为人工miRNA（artificial microRNA, amiRNA），可由聚合酶Ⅱ或聚合酶Ⅲ启动子转录^[12]^，是利用天然miRNA作用原理设计的，以一个或多个特定基因为靶标的小RNA分子，能够高效、特异地抑制基因的表达。在拟南芥中，基于amiRNA的高特异性基因沉默通过结构诱导或组织特异性聚合酶Ⅱ启动子^[13]^得以应用。在哺乳动物中，Hannon和Elledge构建了以小分子RNA为基础的大型shRNA载体文库，这些载体的靶点涉及人类和小鼠的很多基因；他们观察到了比以前的shRNA文库更为有效的基因抑制现象，促进了基因功能的研究。

### 2.3 适用于哺乳动物的siRNA文库

在哺乳动物细胞中，长dsRNA往往触发非特异性免疫，导致序列非特异性的蛋白表达下调，转变为长度超过30 bp的非自身dsRNA，因此，siRNA（通常为21 bp）已经成为诱导哺乳动物RNAi的主要形式。在哺乳动物中的RNAi筛选开始于肿瘤坏死因子相关的凋亡诱导配体（tumor necrosis factor related apoptosis inducing ligand, TRAIL）介导细胞凋亡的调控子的确认，TRAIL是TNF家族的一员，具有抗肿瘤的功能。Sonnichsen等^[10]^应用Dharmacon公司生产的针对510个基因的siRNA文库转染HeLa细胞，筛选出对TRAIL诱导细胞凋亡有调控作用的基因。Sonnichsen等^[10]^的筛选对了解TRAIL的作用机制和抗肿瘤药物的开发有重要的意义。

目前构建siRNA文库的方法主要有合成化学法和酶学工程方法，化学合成方法利用合成siRNA在高通量遗传筛选方面进行研究，已常规用于哺乳动物细胞中。研究人员还发现了大量与癌症功能、干细胞生物学和病毒感染有关的基因，促进人们进一步了解这些基因的病理生理过程。为了解决化学合成试剂和转染试剂耗费大量费用的问题，研究者发现了酶学工程方法合成的siRNA（enzymatically prepared siRNA, esiRNA）文库^[14]^。EsiRNA文库起源于DNA模板（cDNA文库或携带目的DNA的质粒文库）并且被加工进入针对同一个mRNA序列的不同siRNA中，相对于化学合成siRNA文库，效率更高。目前研究者将创建esiRNA文库研究那些基因组还未被测序的生物。

化学合成的和esiRNA文库的共同问题是转染试剂伴随大量的毒性反应。并且由于文库的转染效率很低，均不适用于原代细胞的筛选。因此，上述文库均无法很好地应用于哺乳动物细胞中，而病毒载体的出现，有可能克服上述的难题^[15]^。

## 3 哺乳动物中由不同病毒载体介导的RNAi文库

### 3.1 腺病毒文库

腺病毒是一种无包膜的双链DNA病毒，编码基因组中分子量超过36 kb的40多种蛋白。腺病毒相对安全，易于扩增。目前腺病毒文库已经广泛的用于基因治疗的临床试验中^[15]^，甚至被国内批准用于一些特定的肿瘤治疗中，如*p53*基因的抗癌治疗^[16]^。近来有复制缺陷的腺病毒载体用于传递编码基因的蛋白质或siRNA^[17]^。SilenceSelect文库就是基于带有shRNA的腺病毒载体^[18]^，可用来沉默与人类疾病有关的药物靶点。

腺病毒基因组相对较大，其宿主蛋白与载体间的非特异性干扰等原因限制了这种病毒在文库筛选方面的应用。除此之外，腺病毒载体高表达的非编码RNA，病毒相关RNA1和RNA2，可以作为竞争性底物使Dicer和RISC饱和，抑制RNAi通路，最终通过RNAi沉默机制进行干扰^[19]^。第三代腺病毒载体，其所有的病毒编码序列都被非编码DNA和复合序列所取代，理论上避免了上述这些问题^[20]^，但由于在载体的生成和加工方面的技术问题，并未得到大规模的应用。最为关键的问题是，腺病毒采用游离基因传递的方法只能引起瞬时转基因表达。然而，研究者始终认为：腺病毒RNAi文库依然是体内研究的一个重要选择。

### 3.2 逆转录病毒文库

逆转录病毒，比如基于Moloney小鼠白血病的病毒^[21]^，可以整合进入宿主细胞基因组，甚至可以长期在快速分裂的细胞中进行转基因表达，相对于腺病毒文库的瞬时转基因表达是一个相当大的优势。因此，逆转录病毒文库可以应用于RNAi领域中。Jensen等^[22]^从逆转录病毒文库中筛选出高功能性和低功能性的shRNA茎环，对shRNA的设计有很重要的指导意义。然而，逆转录病毒文库的不足之处是：转染效率很低，只能转染分裂期细胞，对于慢分裂细胞或有丝分裂后期的细胞，如神经元细胞，无法转染。

### 3.3 慢病毒文库

慢病毒载体从伪膜带有VSV-G包膜蛋白质的人类1型免疫缺陷病毒中加工而来，与逆转录病毒和腺病毒载体相比，它的优势在于可以转染分裂期和非分裂期的细胞，容纳外源性基因片段大，可以长期稳定表达。由于慢病毒载体广泛的趋向性，可以整合入宿主细胞的基因组并在大多数细胞中高效转染，不产生任何有效的细胞免疫应答，介导的转基因表达能持续数月，在RNAi文库中应用广泛。最近由ThermoScientific、Cold Spring Harbor Library与美国哈弗大学合作开发了两种慢病毒RNAi文库，两者的设计都基于miR-30-shRNA，分别用于shRNA的组成性表达和诱导性表达^[23]^。

由以上内容可知，逆转录病毒或慢病毒载体的RNAi文库在体外的应用相对简单，但是在体内的应用仍然处于瓶颈期。节约且可行的方法是在异种植皮术的动物中进行干细胞或癌细胞的体外转导^[24]^，或在体外转导胚胎干细胞，产生组织特异性或全身性的RNAi的转基因老鼠^[25, 26]^。然而，人们仍无法实现基因到基因的高通量筛选，也无法直接应用于体内，而这些仍需要研究人员进一步的探索。

## 4 RNAi文库在肿瘤研究中的应用

### 4.1 寻找新型抗肿瘤药物靶点中的应用

肿瘤治疗的原则是阻止肿瘤细胞增殖，或使肿瘤细胞生长受到抑制甚至产生直接的细胞自杀性行为。而RNAi文库就是利用细胞活力测定筛选出具有抑制肿瘤细胞增殖能力的细胞，进而发现抗肿瘤通路上的重要靶点。基于病毒载体介导的RNAi筛选系统特别适合在人类肿瘤细胞中进行基因功能分析，并且已经成功的发现了一些肿瘤抑制基因，这些基因的抑制会导致肿瘤增殖能力的增高以及相关支持物的增长。两个类似的研究从shRNA文库中筛选出一些基因，研究人员发现，这些基因能够在人类肿瘤细胞系（结肠癌细胞系DLD-1和HCT116，乳腺癌细胞系HGC1954、MCF-10A、MDA-MB-435、MDA-MB-231、ZR-75.1和T47D）和正常乳腺上皮细胞中促进细胞增殖，不仅如此，有一些基因亚型也是生长和增殖所必须的。上述结果均表明这些基因很有可能成为新型抗癌治疗靶点^[27]^。为了研究酪氨酸激酶（tyrosine kinases, TKs）对细胞生长的抑制作用，研究人员在乳腺癌细胞系中利用酪氨酸激酶特异性的siRNA构建了一个siRNA文库，结果表明：TKs以及大约90余种蛋白在乳腺细胞信号转导通路中发挥重要的作用，包括细胞的增殖，凋亡，分化及细胞的运动能力等^[28]^。

### 4.2 逆转肿瘤耐药研究中的应用

目前，治疗恶性肿瘤的主要手段包括化学治疗及放射治疗，而影响化疗效果的一个重要原因是肿瘤细胞产生了对化疗药物的耐药性。肿瘤细胞的耐药性，尤其是多药耐药问题，是目前的研究重点之一。Lai等^[29]^在哺乳细胞中利用能改变复杂细胞表型的siRNA构建了一个随机siRNA文库，在结直肠癌细胞中发现了一系列siRNA编码的基因，这些基因能在氟尿嘧啶或肿瘤坏死因子（tumor necrosis factor, TGF）-α介导的细胞死亡机制中，逆转肿瘤细胞的耐药性。他莫昔芬可以阻断乳腺癌雌激素受体α（estrogen receptor α, Erα）信号传导通路，是治疗乳腺癌最常用的药物之一，但由于耐药性的出现，限制了它的应用。Iorns等^[30]^采用针对779个激酶和相关蛋白的RNAi文库，发现依赖磷酸肌醇激酶1（phosphoinositide kinase 1, PDK1）信号通路对于多个ERα拮抗剂均有很强的敏感性，这为发现他莫昔芬治疗的新靶点，进一步探索乳腺癌细胞的耐药性提供了新的方向。紫杉醇是临床上常用的治疗肿瘤的化疗药物，然而，乳腺癌、卵巢癌和非小细胞肺癌等肿瘤常常对紫杉醇产生耐药。Xu等^[31]^建立了针对人类基因组的慢病毒siRNA文库，他们发现：septin10（SEPT10）的表达水平与人类对紫杉醇的敏感性正相关，这预示着SEPT10很有可能成为紫杉醇耐药相关的生物标志物，这对于开辟基因治疗和化学治疗的新方法有重大的作用。

### 4.3 探索肿瘤迁移及转移研究中的应用

目前，肿瘤迁移、转移已成为当前肿瘤治疗中亟待解决的重要问题，近年来研究人员通过对肿瘤迁移、转移分子机制的深入研究和了解，已为肿瘤的治疗和防治提供了新思路和新靶点。为了研究局部粘着斑激酶（focal adhesion kinase, FAK）与细胞增殖、粘附、迁移的关系，Tsutsumi等^[32]^构建了由质粒表达的shRNA文库，在Hela和HT1080细胞中挑选出能抑制FAK表达的克隆。他们发现：FAK蛋白表达下调能够抑制细胞的粘附、迁移和增殖能力，在肿瘤形成和生长过程中起着重要的作用。为了探索*CICP1*基因在肿瘤转移中发挥的作用，Nagai等^[33]^构建了稳定的肺癌转移细胞系——LNM35的RNAi克隆，实验结果表明：CICP1及其配体SEMA4B在体外能够显著抑制细胞的运动能力，在体内可以抑制肺癌细胞的转移能力。这预示着CICP1及其配体SEMA4B将有可能在未来成为抑制肺癌转移的分子治疗靶点。

### 4.4 指导肿瘤诊断和治疗中的应用

近年来，肿瘤的诊断、治疗均取得了显著的进步，这反映了肿瘤研究领域取得的卓越成绩。肿瘤病研究的最终目的就是解决肿瘤诊断和治疗中的难题。而RNAi文库可以针对基因起作用，为其可应用于肿瘤的诊断和治疗提供了机会。Bjorkman等^[34]^利用基于细胞芯片的siRNA文库，解释了肿瘤中已知的和新近发现的表观遗传学酶的作用，极大地促进了肿瘤表观遗传学的研究，在未来将有可能用于肿瘤的诊断和治疗。为了探究急性髓性白血病的治疗的新策略，Zuber等^[35]^利用一个针对已知染色质调定点的shRNA文库，发现溴区包含蛋白4（bromodomain-containing 4, Brd4）对于维持肿瘤的稳定性具有重要作用，无论在体内或体外抑制Brd4的表达都能产生抗白血病的效应。这些结果表明：抑制Brd4的表达很有可能成为急性髓性白血病治疗的方法之一。为了探索肿瘤药物的联合治疗策略，Liu-Sullivan等^[36]^在四种肺癌细胞系中采用一个针对不同肿瘤基因的混合shRNA文库，寻找抑制polo-like kinase（PLK1）联合作用的靶点。他们发现：维甲酸类药物确实能够提高药物敏感性并增强PLK1的抑制作用，进而阻止有丝分裂，导致细胞凋亡。这些结果表明：维甲酸可用来增强PLK1抑制剂——GSK461364的作用，并进一步说明RNAi文库能成为证实联合靶向治疗的有效工具。尤其令人兴奋的是，近期研究结果表明，RNAi文库技术甚至可可用于肿瘤的分子治疗中，进行特异的基因沉默并抑制肿瘤的生长^[37]^。

## 5 展望

各种文库的迅速发展促使研究者不断致力于RNAi的研究，而RNAi技术以及RNAi文库应用的日趋成熟，为人类的各种重大疾病尤其是肿瘤疾病的治疗提供了坚实的理论基础。但RNAi存在的非特异性效应阻碍了RNAi文库的进一步临床应用，主要包括脱靶效应和免疫副反应。RNAi过程中可能伴随的脱靶沉默效应，主要是因为直接导入细胞或胞内由dsRNA分解的siRNA与胞内其它非靶正常基因存在部分序列同源性。而免疫副作用主要是激活天然免疫系统，诱导炎症因子的产生，引起细胞的生长抑制等毒性作用。由脱靶效应产生的意外的基因调控和免疫副反应极大地限制着RNAi文库技术的体内应用，成为其应用的一个主要障碍。如何既避免siRNA对机体免疫系统的脱靶副作用又保持siRNA的特异性靶基因沉默作用，成为siRNA设计的关键。如何构建使已知基因沉默效率较高的，带有较少脱靶效应的，在体内和体外同时具备高通量筛选能力的新型RNAi文库，是研究者需要进一步解决的问题。如何将RNAi文库技术应用于体内研究进而进一步应用于临床，怎样应用于肿瘤病人的个体化治疗，是研究人员和肿瘤科医生共同探索的方向。尽管存在一系列理论和技术上的难题，我们仍坚信，随着RNAi技术以及RNAi文库的不断发展，一定能在肿瘤研究领域开拓广阔的应用前景。
